# Medical device landscape for communicable and noncommunicable diseases in low-income countries

**DOI:** 10.1186/s12992-018-0355-8

**Published:** 2018-07-04

**Authors:** Amir Sabet Sarvestani, Kathleen H. Sienko

**Affiliations:** 1 Design Science Program, University of Michigan, Ann Arbor, USA; 2 Department of Mechanical Engineering, University of Michigan, Ann Arbor, USA; 3 Department of Biomedical Engineering, University of Michigan, Ann Arbor, USA

**Keywords:** Global health, Health technology, Low-income countries, Medical devices, Noncommunicable diseases, Primary health care facilities

## Abstract

**Background:**

This study characterized the landscape of commercially available medical devices specifically designed for use in low-income countries (LICs).

**Methods:**

A state-of-the-art review of peer-reviewed publications, patents, global health databases, and online resources was performed. The criteria established for a health technology’s inclusion in the study were: it met the definition of a medical device; it was designed and developed to address one of the top ten causes of death in LICs, Millennium Development Goal (MDG) 4, or MDG 5; and there was evidence of its commercialization.

**Results:**

Analysis identified 134 commercialized devices exclusively designed for use in LICs. More than 85% of devices were designed to address infectious diseases or child or maternal health (MDG 4 or 5, respectively). None of the identified devices addressed prevention of noncommunicable diseases (NCDs). Only 8% of devices were designed for use in primary health facilities by non-physician health providers.

**Conclusion:**

There is a significant mismatch between the projected global burden of disease due to NCDs and the relevant number of commercialized medical devices designed specifically for use in LICs. A limited number of commercialized devices were designed for use by non-physician health providers. These findings suggest the need for medical devices targeting NCDs in LICs and design processes that consider the broader context of design and engage stakeholders throughout all phases of design.

## Background

The availability, accessibility, and effectiveness of medical devices are vital in achieving the highest quality of care within health systems [1]. Medical devices, defined as “articles, instruments, apparatus, or machines that are used in the prevention, diagnosis, or treatment of illness or disease, or for detecting, measuring, restoring, correcting, or modifying the structure or function of the body for some health purpose” [2], are a major part of health technologies (which also include vaccines and medicines), and an essential building block in any functioning health system [1]. The World Health Organization (WHO) indicates that there are over 10,000 types and brands of medical devices globally, ranging from basic stethoscopes to complex diagnostic imaging machines; it estimated that the global medical devices market was over $350 billion in 2011 [3]. However, historically, the overwhelming majority (~ 90%) of health technology sales have occurred within high- and middle-income countries [3, 4].

Almost 80% of medical devices in LICs are acquired by donation [5]. In addition to donations, medical devices are also acquired through technology transfer: local production of devices that resemble technology designed for use in high-income countries (HICs) or the low-cost sale of older models of devices originally designed for use in HICs [5, 6]. However, use of medical devices in LICs that were originally designed for use in HIC are not entirely successful; one study noted that 40% of medical devices were dysfunctional in LICs versus less than 1% in HICs [6, 7]. In LICs, constraints including unreliable energy supply and water, limited distribution and infrastructure, inadequate or untrained workforces, lack of spare parts, required consumables, and high costs affect the availability and acceptability of many devices [8].

Decades of investing in lifesaving medical devices, training health care providers at various levels, and planning strategic interventions globally have led to drastic reductions in mortality due to infectious diseases, maternal and child illness, and malnutrition [9]. While infectious diseases have been in the spotlight for the last few decades, noncommunicable diseases (NCDs) accounted for 63% of global deaths in 2008–80% of which occurred in low- and middle-income countries (LMICs) [10]. In fact, deaths from NCDs were projected to increase to 52 million by 2030, with NCDs in LMICs responsible for three times as many disability-adjusted life years (DALYs) and nearly five times as many deaths as infectious diseases, maternal, perinatal, and nutritional conditions combined [10]. Given these projections, the WHO and the UN called for “25 by 25”, i.e., a 25% reduction in the mortality caused by NCDs among individuals between 30 and 70 years of age by 2025 [11, 12].

Even though the role of medical devices in addressing pressing global health challenges is widely acknowledged, the landscape of commercialized medical devices specifically designed for LICs is unknown [3]. For instance, it is essential to know the availability of devices to address NCDs, and to evaluate the number of devices designed for use by non-physician health providers, given the limited number of highly trained human resources in LICs (47% of the WHO member states reported having less than 1 physician per 1000 population [13]). This research characterizes the current landscape of commercialized medical devices that are specifically designed for use in LICs.

## Methods

A state-of-the-art review [14] was conducted by a team of 30 research assistants from the University of Michigan to identify health technologies designed to address at least one of the top ten causes of death, UN Millennium Development Goal (MDG) 4 (reduce child mortality), or MDG 5 (improve maternal health) in LICs. The research team searched online data only. The information sources included the WHO’s compendium of innovative health technologies for low-resource settings [15] and MANDATE’s technology assessment tool [16]. Also, peer-reviewed, published articles and patent applications were identified using PubMed, Google Scholar, and the US Patent and Trademark Office database. Keywords used to identify devices included, but were not limited to, the following terms, individually or in combination: medical devices, health technology, frugal design, engineering design, global health, low-income countries, low- and middle-income countries. Names of specific health challenges (e.g., cancer) in conjunction with other keywords were also used as search terms.

Following a general search for health technologies meeting these preliminary criteria, a subset of the identified health technologies was classified as medical devices and considered for this study based on the following inclusion criteria:

1. Met the definition of a medical device [2], and
2. Designed specifically to address a health challenge in LICs, based on the intentions expressed by the developer of the medical device, and
3. Supplied evidence to support the commercialization (i.e., available and accessible to end-users in target settings [17]) of the device in LICs.

A template was developed to systematically compile information about each identified device including scope (diagnostic, prevention, and treatment), stage (concept, clinical trial, commercialized), developer, testing and implementation details, funding sources, and references. Categorizing the devices by scope and stage allowed for additional analysis on the number of available devices. The data for devices selected for inclusion were then reviewed for accuracy and assessed for bias by two study team members.

The data collected during this study were subsequently included in an online, collaborative, open-access platform, known as the Compendium of Medical Devices for Global Health [18]. This information is currently available for public access. The findings and interpretation presented here are based on the data collected between January 15, 2010 and December 31, 2013.

## Results

**Fig. 1:**
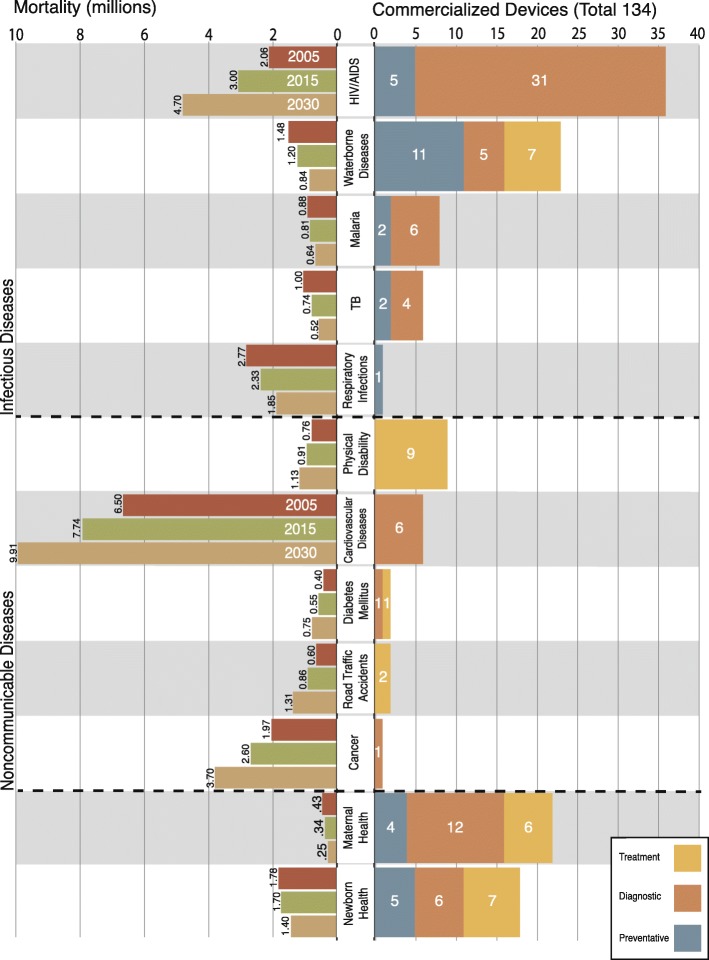
The left side bar graphs show the actual (2005) and projected (2015 and 2030) mortality in LICs [19]. The right side bar graphs show the commercialized medical devices designed to address one of the top 10 causes of death or MDG 4 or MDG 5 in LICs. Devices are categorized based on the health problem addressed and their scope

The initial search identified 401 health technologies, inclusive of medical devices, mHealth solutions, and vaccines, intended for use in LICs. The subsequent classification process resulted in the identification of 358 medical devices of which 134 (37% of total) met the “commercialization” criterion (inclusion criteria #3) to address specific health topics in LICs. One imaging device was double-counted because it is used for both maternal and infant health. The remaining 224 devices were either at the early concept (prototype) design stage or at the clinical trial stage. Fig. 1 shows the number of devices developed per health topic, clustered based on their associated scope (i.e., diagnostic, prevention, and treatment), and mortality projections for infectious diseases, NCDs, pregnancy-related complications, and newborn conditions in LICs [19]. Of the total 134 commercialized devices, 114 (85%) targeted infectious diseases or MDG 4 or MDG 5. The HIV/AIDS topic had the largest number of commercialized devices (36), with the majority in the diagnostic category (31). There was one commercialized device for the respiratory infections topic and only one for the cancer topic. Other than for the waterborne diseases topic, there was only one device-based treatment for infectious diseases.

**Fig. 2:**
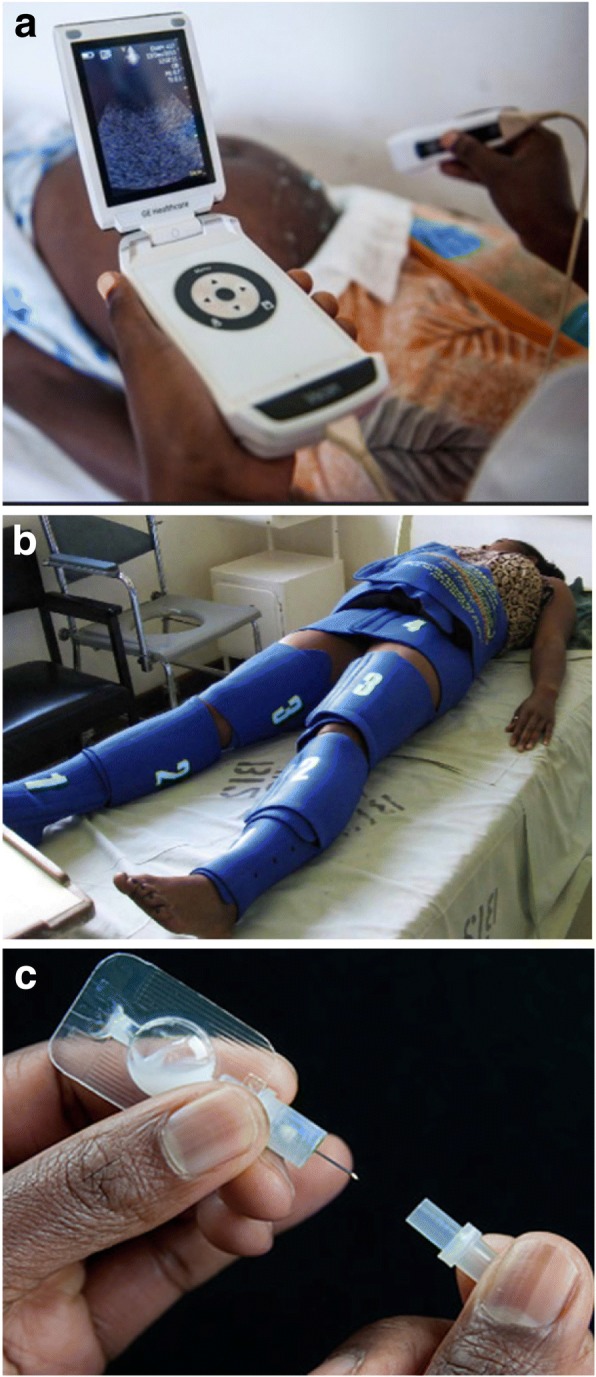
**a** Vscan used in a health post in sub-Saharan Africa (image from http://www.gesustainability.com); **b** LifeWrap applied on a woman (image from http://www.lifewraps.org); **c** Uniject™ (image from http://www.path.org)

Only 20 commercialized medical devices (< 15%) were identified to address NCDs, revealing a significant mismatch between the number of commercialized devices and the increasing global burden of NCDs. For instance, fewer than 10 devices addressed cardiovascular diseases and cancer in LICs, whereas the annual cardiovascular disease mortality was projected to increase by 6 million and the number of annual cancer deaths by 4 million over the next 20 years in LICs [19]. Notably, none of the identified devices were designed for the prevention of NCDs. Vscan (Fig. 2a), a pocket-size, portable, and low-cost ultrasound machine is an example of a medical device that has been designed and commercialized considering the specific needs of LICs [20].

Among the identified devices, only 30 were commercialized for use at the primary health care level, which are typically staffed by non-physician health care providers, and none addressed any of the NCDs. LifeWrap, a fully mechanical device used to control postpartum hemorrhage (Fig. 2b), a leading cause of global maternal mortality [21], and Uniject™, an auto-disable injection device used to deliver vaccines and drugs (Fig. 2c) [22] are examples of medical devices designed for use by non-physician health care providers. These devices are notable because they are relatively easy to learn how to use, do not require consumables, and do not rely on electricity.

From the analysis, 55 commercialized devices were powered mechanically and another 52 devices (mostly diagnostic) employed a chemical reaction, suggesting that approximately 80% did not rely on electricity as a power source.

## Discussion

Medical devices have a limited, yet important role in the effective delivery of health care [3]. The role of medical devices and health technology in the fight against NCDs was emphasized in the Global Action Plan for the Prevention and Control of NCDs proposed by the WHO and endorsed by the World Health Assembly in 2013 [23]. However, the evident mismatch between the number of commercialized medical devices, which are specifically designed for and accessible in LICs, and the projected burden of diseases due to NCDs in LICs is of concern. Even though NCDs are projected to represent the greatest burden on health in the near future, the number of medical devices designed and commercialized to prevent, diagnose, and treat physical disability, cardiovascular diseases, diabetes mellitus, road traffic accidents, and cancer combined is considerably smaller than the number designed and commercialized to prevent, diagnose, and treat infectious diseases, maternal health, and infant health.

There is a critical gap between designing and developing a safe and effective medical device and implementing and scaling that device within the target setting [7]. The total number of concept solutions, early stage prototypes, and low-scale medical devices aimed at addressing global health challenges greatly exceeds the number of medical devices included in this study. Among the hundreds of medical devices designed to be low cost and contextually appropriate that were not included, many likely failed to reach scale because they did not effectively address an unmet need, lacked established pathways to facilitate the transition from technical designers to organizations or individuals skilled in implementation and commercialization of technology, lacked adequate funding, encountered challenges navigating regulatory pathways or securing appropriate intellectual property, or insufficiently managed the supply chain (e.g., procurement, distribution, maintenance) [7, 24, 25]. Overcoming the complexities associated with implementing and scaling a medical device may require modeling and simulation of scale-up, creation of effective delivery mechanisms, pursuit of novel financing, and implementation of evidence-based operational practices [7, 26].

The lack of medical device maintenance, a significant challenge to health systems in LICs, negatively impacts patient care and public health [27]. Factors affecting the maintainability of medical devices include shortages of trained biomedical technicians, limited access to spare parts and consumables, and infrastructural constraints such as consistent power availability [27]. From a design perspective, inclusion of only essential functions, reduction of the number of custom components, and incorporation of maintenance and troubleshooting aids can improve the maintainability of medical devices [7]. From a health care system perspective, the practice of preventative maintenance can extend the useful lifetime of medical devices [27], and local production can increase the likelihood of locally available product support and the availability of medical device consumables [7].

Successful design for LICs also depends on understanding the broader issues associated with implementation in the early stages of the development process rather than after the validation and production stages [28, 29]. For example, considerations regarding medical device commercialization and adoption are likely to be different in LICs [25]. Therefore, novel medical device design frameworks that consider downstream variables (e.g., manufacturing plans, regulatory pathways, etc.) as well as the broader context during the front-end phases of design (e.g., development of product requirements and technical specifications) are needed [28]. Design approaches that consider local and regional constraints, cultural contexts, and stakeholder needs, and enhance the capacity of the local health care workforce are particularly effective [6, 8].

The limited availability of highly trained health providers presents an extraordinary challenge in providing universal quality care. For instance, while Africa bears more than 24% of the global burden of disease, it only has access to 2% of the global physician supply [30]. Therefore, non-physician health care providers such as community health workers have the potential to extend access to essential health services, particularly in rural settings within LICs [31]. Task-shifting promotes the efficient use of available human resources by transferring appropriate tasks typically performed by “highly qualified health workers to health workers with shorter training and fewer qualifications” [32]. To date, limited medical devices have been designed specifically for task-shifting applications; such devices can play a critical role in improving access to universal care and tackling the threat of NCDs, particularly in rural LICs. Devices that are easy to use, have limited components, no need for spare parts, minimal to no maintenance or need for calibration, and use reliable and readily available energy sources may increase their suitability for community health workers performing task-shifting duties [28].

Study limitations included the possible omission of relevant medical devices due to online information that was insufficient, not found, or not in English. Descriptions and information used to classify the identified medical devices were limited by the information provided by the device designers and developers, and other available online sources, and the study team did not independently evaluate and validate these claims. This study solely focused on identifying medical devices designed for and commercialized in LICs. Of course there are other devices used in these settings that might not fit the inclusion criteria, but are still effective in addressing health challenges. The focus on actual and projected mortality due to infectious diseases, NCDs, pregnancy-related complications, and newborn conditions in LICs did not consider the DALYs, a limitation which potentially could skew the outcomes, since some devices could have improved the life conditions of affected individuals (projected as DALYs).

## Conclusions

The magnitude of the NCD epidemic illustrates the need for targeted medical device development, considering the context and end-user environment of use. The mismatch between the number of commercially available medical devices and the projected global burden of disease, as well as the limited number of available devices designed for use by community health workers to support task-shifting will require policymakers and the global health community to provide intellectual, financial, and regulatory support in order to develop the necessary technology in a timely manner. Although it is not possible to separate the effects of global health technologies, in this case, medical devices for LICs, from the effects of social, political, economical, and healthcare measures on mortality in LICs [7], availability and accessibility of medical devices are important and if part of a comprehensive solution, can positively impact global mortality and morbidity trends.
